# Differences in the oral and intestinal microbiotas in pregnant women varying in periodontitis and gestational diabetes mellitus conditions

**DOI:** 10.1080/20002297.2021.1883382

**Published:** 2021-02-09

**Authors:** Xin Zhang, Pei Wang, Liangkun Ma, Rongjun Guo, Yongjing Zhang, Peng Wang, Jizhi Zhao, Juntao Liu

**Affiliations:** a Department of Stomatology, Peking Union Medical College Hospital, Chinese Academy of Medical Sciences, Peking Union Medical College, Beijing, China; b Department of gynaecology and obstetrics, Peking Union Medical College Hospital,Chinese Academy of Medical Sciences, Peking Union Medical College, Beijing, China; c Beijing Hospital, National Center of Gerontology, Institute of Geriatric Medicine, Chinese Academy of Medical Sciences, Beijing China; d Novogene Bioinformatics Technology Co., Ltd., Tianjin, China

**Keywords:** Periodontitis, gestational diabetes mellitus, oral microbiota, intestinal microbiota, pregnant

## Abstract

**Background**: The present study aimed to investigate the potential association between oral and intestinal microbiotas of pregnant women with periodontitis and/or gestational diabetes mellitus (GDM) in the second trimester.**Methods**: Four groups were defined: periodontitis (n = 28), GDM (n = 7), periodontitis + GDM (n = 7), and periodontitis- and GDM-free controls (n = 27). The oral and intestinal microbiomes were analyzed using the 16S rRNA sequencing technique.**Results**: Periodontitis alone significantly decreased the oral microbial diversity (by Shannon index, p = 0.003) and changed the structure of the oral microbial community (by AMOVA, p 0.001). GDM alone significantly increased the oral microbial diversity (by Shannon index, p = 0.049), and when combined with periodontitis, GDM significantly decreased the intestinal microbial richness (by observed species, p = 0.018) and influenced the structure of intestinal microbial community (by AMOVA, p = 0.043). The differentially abundant microbial taxa among different groups in both oral and intestinal samples were identified by LEfSe analysis, and limited taxa showed consistent trends. The numbers and ratios of oral-intestinal shared operational taxonomical units were the least in the periodontitis + GDM group.**Conclusions**: A close relationship between the oral microbiota and pregnant periodontitis was shown. Significant changes occur in both the oral and intestinal microbiomes when periodontitis was coupled with GDM. A separate influence of periodontitis and GDM on the oral and intestinal microbiotas may be indicated.

Periodontitis is one of the most common infectious diseases in periodontal tissues. In addition to gingival inflammation, it involves inversible deeper supportive tissue destruction and is one of the main reasons for adult tooth loss [1]. A close relationship was reported between periodontal disease and pregnancy health [2]. A higher prevalence and greater severity of periodontal inflammation occur during pregnancy than during prepregnancy [3]. Gestational diabetes mellitus (GDM) is a common complication in pregnant women. It is defined as diabetes or any degree of glucose intolerance that occurs during pregnancy in a woman who has no diabetes or glucose intolerance before pregnancy [4]. Both periodontitis and GDM are associated with adverse pregnancy outcomes and play roles in pregnancy health [2,5]. In addition, correlations have been shown between periodontitis and GDM [6,7]; it was reported that periodontitis might predispose individuals to the development of GDM, and a synergistic effect was found with GDM in the development of preeclampsia [8].

The human microbiome is regarded as our second genome. It resides in the human body and plays roles in host metabolism, immunity, and overall health [9]. Microbial disturbance may be the reason for local and systemic disorders and the result that was adapted with disease status. The gastrointestinal tract and oral cavity are two important parts of the human digestive system and harbor two main microbiomes in the body. They might interact with each other and play roles in human health and disease [10,11]. Significant variations have been observed in the oral bacterial communities of periodontitis patients in relation to periodontal health [12–14]. This is not surprising when considering the polymicrobial infection nature of periodontitis. Few studies have focused on the relationship between GDM and oral microbiota, and various degrees of changes in GDM-related oral microbiotas have been reported [15–18]. Differences in the intestinal microbiomes between GDM patients and healthy controls have been explored by an increasing number of studies, and most of them have reported an influence of GDM on the intestinal microbiota [17–20]. To date, the association between pregnant periodontitis and intestinal microbiota remains unclear. In addition, in the majority of the studies investigating the effects of GDM on oral and intestinal microbiota, the periodontal health status of the participants has not been described. Therefore, within the definition of GDM, two conditions, GDM with and without periodontitis, could be considered. The problems of whether a combined effect exists in periodontitis and GDM on oral microbiota, whether the influence of GDM on intestinal microbiota is dependent or independent of periodontitis, and whether crosstalk between oral and intestinal microbiota is associated with periodontitis and GDM status need to be further explored.

In the present study, the oral and intestinal microbiomes of second-trimester pregnant women assigned to four groups, defined as periodontitis, GDM, periodontitis + GDM and healthy controls, were assessed via 16S rRNA gene amplicon sequencing of the V4 region. The relationship between the oral and intestinal microbiotas and periodontitis and GDM, as well as the association of periodontitis and GDM, were further explored.

## Materials and methods

### Study populations

This study was based on a whole-pregnancy follow-up cohort (The gastrointestinal microbial cohort study during pregnancy). All participants were recruited at their first visit before 14 weeks of gestation and monitored until delivery. All the subjects were aged from 20 to 45 years. Women who had digestive system diseases; metabolic diseases including hypertension, diabetes and dyslipidemia; or immune system diseases and tumors; as well as women who had taken antibiotics or probiotics within the preceding 3 months; were excluded. Sixty-nine pregnant women in the second trimester (20–28 weeks) who were nonsmokers and not affected by other pregnancy conditions besides GDM were included in this study. They all consented to receive oral examinations and provided fecal and oral samples. Sixty-nine paired salivary and fecal samples, 61 supragingival plaque samples and 59 subgingival plaque samples were contributed. This study was conducted with the informed consent of all participants and was approved by the Ethics Committee of Peking Union Medical College Hospital (JS-1535).

### Oral examination and data collection

A full-mouth oral examination was conducted by a single experienced dentist for each patient. Periodontal parameters of probing depth (PD) and bleeding index (BI) and carious parameters of decayed, missing, and filled teeth (DFMT) were recorded. Attachment loss was calculated at each site with a gingival recession based on gingival recession distance and PD. Periodontitis was defined as ≥2 interproximal sites with AL ≥3 mm and ≥2 interproximal sites with PD ≥4 mm (not on same tooth) or one site with PD ≥5 mm [21]. Participants who did not meet the criteria of periodontitis were grouped as non-periodontitis. GDM was defined using established criteria from the International Association of Diabetes and Pregnancy Study Groups (IADPSG). A standard 2 h, 75 g oral glucose tolerance test (OGTT) was performed at 24–28 gestational weeks. Pregnant women were diagnosed with GDM if one or more glucose levels were elevated: fasting ≥ 5.1 mmol/L, 1 h ≥ 10.0 mmol/L, 2 h ≥ 8.5 mmol/L. Information on maternal age, prepregnant BMI, gestational weight gain and levels of fasting blood glucose (FBG) were obtained from the medical records.

### Sample collection

The fecal samples were collected at home between 24–28 gestational weeks. The samples were stored in collection tubes (Stratec Corporation, Germany) at room temperature. Salivary samples were collected at the clinic at 8–10 am according to a well-established protocol. The participants were asked not to brush their teeth on the sampling day. After rinsing the mouth, approximately 1.5 mL of unstimulated saliva was collected from each patient into a 5-mL sterile Eppendorf tube. Dental plaque samples were collected after each oral examination. Pooled plaque samples were collected from the mesiobuccal surfaces of teeth 16, 17, 11, 26, 27, 36, 37, 31, 46, and 47 for supragingival and subgingival samples, respectively. The selected teeth were isolated with cotton rolls and gently air-dried; sterile Gracey curettes were used to scale the plaques and transfer them into sterile Eppendorf tubes each containing 2 mL sterile saline solution. All the samples were transported on ice to the laboratory and then kept frozen at −80°C until use.

### DNA extraction, amplification, and sequencing

Genomic DNA from the samples was extracted using the CTAB method. The quality of the extracted DNA was monitored on 1% agarose gels. The V4 hypervariable regions of the bacterial 16S rRNA gene were amplified using specific primers (16S V4: 515 F-806 R) with barcodes. All PCRs were carried out with a Phusion® High-Fidelity PCR Master Mix (New England Biolabs). Amplicons were extracted from 2% agarose gels and purified using a Qiagen Gel Extraction Kit (Qiagen, Germany). Sequencing libraries were generated using a TruSeq® DNA PCR-Free Sample Preparation Kit (Illumina, USA) following the manufacturer’s recommendations, and index codes were added. The library quality was assessed on the Qubit@ 2.0 Fluorometer (Thermo Scientific) and Agilent Bioanalyzer 2100 system. Then, the library was sequenced on an Illumina HiSeq 2500 platform, and 250-bp paired-end reads were generated.

### Processing of sequencing data

Paired-end reads were assigned to samples based on their unique barcodes and each truncated by cutting off the barcode and primer sequence. Paired-end reads were merged using FLASH (V1.2.7, http://ccb.jhu.edu/software/FLASH/), and the splicing sequences were called raw tags. Quality filtering of the raw tags was performed under specific filtering conditions to obtain high-quality clean tags according to the QIIME (V1.9.1, http://qiime.org/index.html) quality control process. Chimera sequences were then detected and removed with the help of the UCHIME algorithm (UCHIME Algorithm, http://www.drive5.com/usearch/manual/uchime_algo.html) to obtain effective tags [22].

All of the effective tags were clustered into operational taxonomical units (OTUs) using Uparse software (Uparse v7.0.1001, http://drive5.com/uparse/)[23] at 97% sequence similarity and were annotated against the Silva132 database (http://www.arb-silva.de/). Multiple sequence alignment was conducted using MUSCLE software (Version 3.8.31). The OTU abundance information was normalized using a standard sequence number corresponding to the sample with the fewest sequences. All normalized sequences were generated for the downstream analysis.

### Bioinformatics analysis

Four kinds of samples, saliva, supragingival plaque, subgingival plaque and feces, were used in this study and are described by the letters A, B, b and C, respectively. G stands for GDM patients, and H stands for women who were GDM-free. Number 1 indicates women without periodontitis, while number 2 indicates periodontitis. When data of oral samples from saliva, supragingival plaque and subgingival plaque were pooled together, letter O was used to represent the oral source. When comparisons were made between or among groups, *p* < 0.05 was considered to be significant (in two-sided tests).

**Figure 1.**
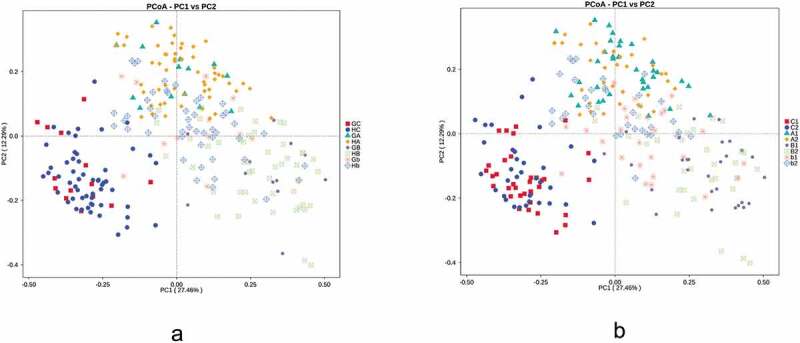
Principal coordinate analysis of the oral and intestinal microbiomes based on weighted UniFrac distance when grouped by GDM (a) and periodontitis (b).GC:GDM+. intestinal, HC:GDM-. intestinal, GA: GDM+. salivary, HA: GDM-. salivary, GB: GDM+. supragingival, HB: GDM-. supragingival, Gb: GDM+. subgingival, Hb: GDM-. subgingival, C1: periodontitis-. intestinal, C2: periodontitis+. intestinal, A1: periodontitis-. salivary, A2: periodontitis+. salivary, B1: periodontitis-. supragingival, B2: periodontitis+. supragingival, b1: periodontitis-. subgingival, b2: periodontitis+. subgingival. Comparisons were made among groups, significance was tested using AMOVA analysis

Principal coordinate analysis (PCoA) based on weighted UniFrac distances was used to investigate the differences in bacterial community structures among various groups under conditions defined by both GDM and periodontitis (Figure 1). PCoA was performed using the ade4 and vegan packages and visualized using ggplot2 in R software. The differences in bacterial community structures between groups were tested by AMOVA using the amova function in mothur software [24]. (Tables S1 and S3).

**Figure 2.**
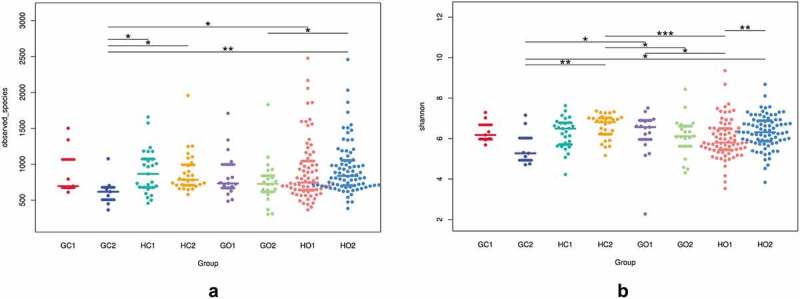
Comparisons of alpha diversity indexes of Observed_species (a) and Shannon (b) among groups defined by GDM and periodontitis.GC1: GDM. intestinal, GC2: periodontitis+GDM. intestinal, HC1: healthy control. intestinal, HC2: peiodontitis. intestinal, GO1: GDM. oral, GO2: periodontitis+GDM. oral, HO1: healthy control. oral, HO2: periodontitis. oral. The data were analyzed with the Wilcoxon rank sum test and are expressed as the median with the interquartile range. * *p*< 0.05, **p < 0.01 ***p < 0.001

Alpha diversity was calculated on the basis of the gene profile for each sample based on the observed species and Shannon indexes. Both indexes were calculated with QIIME (Version 1.9.1) and displayed with R software (Version 2.15.3). The Wilcoxon rank sum test was used to compare the differences between groups (Figure 2).

**Figure 3.**
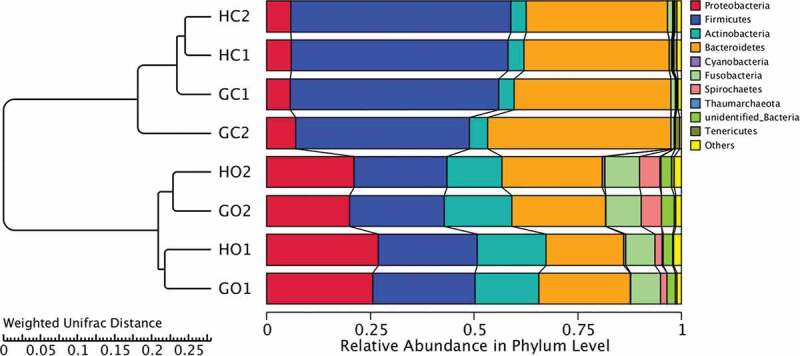
UPGMA analysis based on weighted UniFrac distances with the result of the clustering tree shown on the left and the distribution diagram of the top 10 phylum abundances shown on the right. GC1: GDM. intestinal, GC2: periodontitis+GDM. intestinal, HC1: healthy control. intestinal, HC2: periodontitis. intestinal, GO1: GDM. oral, GO2: periodontitis+GDM. oral, HO1: healthy control. oral, HO2: periodontitis. oral. Comparisons of community structures among groups were performed using AMOVA analysis

The sequential clustering method of the unweighted pair-group method with arithmetic mean (UPGMA) based on weighted UniFrac distances was performed to study the similarity of different bacterial communities. The clustering result was demonstrated in combination with the distribution diagram of the top 10 phylum abundances (Figure 3). The comparison of the top 12 phylum abundances between groups was then performed by MetaStat analysis [23] (Figure S1). These analyses were all performed by R software (Version 2.15.3).

**Figure 4.**
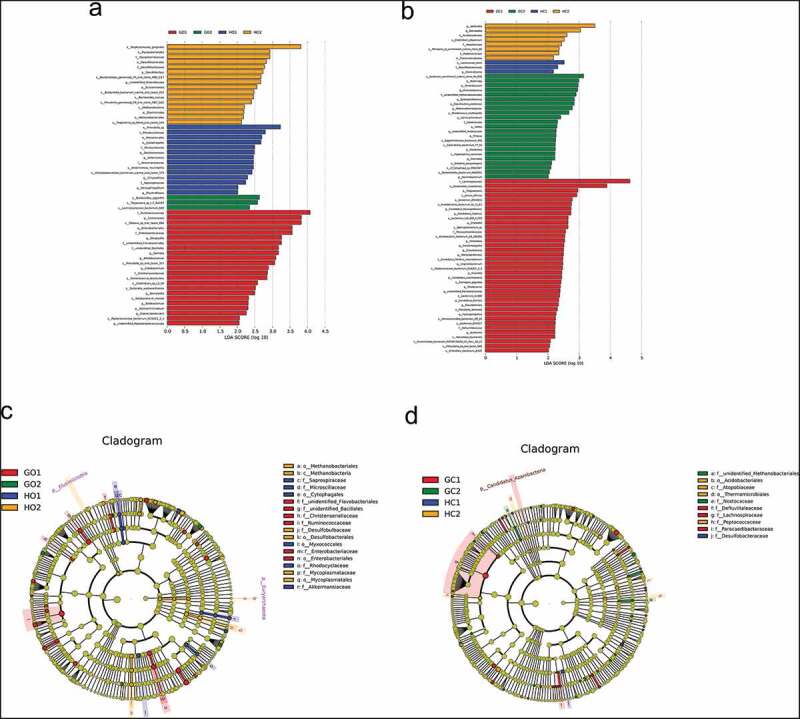
The differentially expressed taxa identified by LEfSe analysis among groups. (a) and (c) were for oral samples and (b) and (d) were for intestinal samples. (a) and (b) demonstrated the differentially abundant bacterial clades in groups of GDM (red), periodontitis (orange), periodontitis +GDM (green) and healthy controls (blue). Clades had a linear discriminants score > 2 and were statistically significant (*p* < 0.05). The circle from the inside to the outside in (c) and (d) represents the classification level from phyla to genera. The diameters of the circles represent the relative abundance. Red, orange, green and blue indicate enrichment in groups of GDM, periodontitis, periodontitis +GDM and healthy controls respectively

The linear discriminant analysis (LDA) coupled with effect size (LEfSe, http://huttenhower.sph.harvard.edu/galaxy) method was used to identify statistically significant biomarkers among groups [25]. The threshold of the logarithmic LDA score for discriminative features was >2.0. A high LDA score indicated a significantly higher abundance of certain taxa (Figure 4).

**Figure 5.**
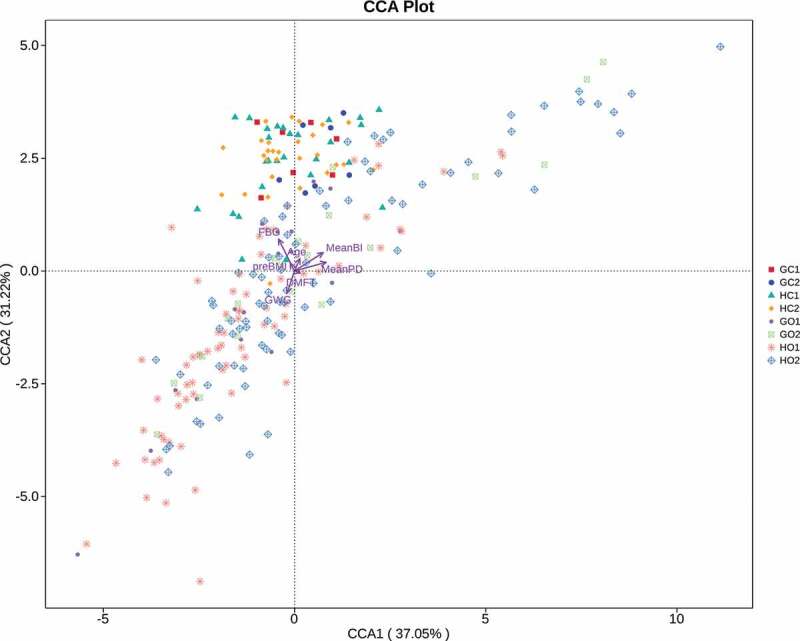
Canonical correlation analysis (CCA) of the oral and intestinal microbial structures, FBG levels and periodontal parameters. Factors of maternal age, preBMI, GWG, FBG levels, DMFT, meanBI and meanPD were included. GC1: GDM. intestinal, GC2: periodontitis+GDM. intestinal, HC1: healthy control. intestinal, HC2: periodontitis. intestinal, GO1: GDM. oral, GO2: periodontitis+GDM. oral, HO1: healthy control. oral, HO2: periodontitis. oral. The significance was tested by CCAenvfit analysis

Canonical correspondence analysis (CCA) was performed using the package ‘vegan’ to evaluate the association between maternal parameters and microbial distributions [26] (Figure 5). The clinical factors were fitted to the ordination plots using the ‘envfit’ function of the vegan package in R (Table S4).

Venn diagrams of the unique or shared OTUs between different groups at the genus level were drawn using R software (Version 2.15.3) (Figure S2).

## Results

1. **Overview of the oral and intestinal microbial compositions and their relationship to periodontitis and GDM**

Among those 69 pregnant women, 35 were diagnosed with periodontitis (7 with accompanying GDM and 28 GDM-free), and 34 were non-periodontitis (7 with accompanying GDM and 27 GDM-free).

A combined weighted UniFrac analysis showed a clear separation between intestinal microbiota and oral microbiota. Although salivary, supragingival and subgingival microbiotas partially overlapped, obvious differences among the three floras were also demonstrated (Figure 1). Significant differences were defined between any two groups from different body sites by AMOVA analysis (*p* < 0.05, Table S1). When defined by GDM, no significant separation was found between groups with and without GDM in any of the four habitats. When defined by periodontitis, a difference was found between the periodontitis group and the non-periodontitis group in both subgingival samples and supragingival samples. The differences in subgingival samples were significant by AMOVA analysis (*p* = 0.001, Table S1). Samples with periodontitis were located higher than those without periodontitis. In salivary and intestinal samples, no clear differences were found.

2. **Comparisons of microbial characteristics among four groups defined by periodontitis and GDM status**

To obtain a comprehensive view of changes in oral and intestinal microbiotas related to periodontitis and GDM, data of oral samples from saliva, supragingival and subgingival microbiomes were grouped together. All samples were defined by periodontitis and GDM. Four groups, periodontitis, GDM, periodontitis + GDM and healthy controls, were obtained for oral and intestinal samples, respectively. The demographic and clinical parameters are summarized in Table S2. These four groups differed significantly in FBG levels and periodontal parameters of meanPD and meanBI (*p* < 0.05). In addition, the ages of the women with GDM (including the GDM group and the periodontitis + GDM group) were significantly older than those without GDM (*p* = 0.022).

The alpha diversities of the oral and intestinal microbiotas were compared among groups (Figure 2). Values of the observed species index for both oral and intestinal samples in groups with GDM were lower than in those without GDM. The differences between periodontitis + GDM group and healthy controls and between periodontitis + GDM and periodontitis groups in intestinal samples and those between periodontitis + GDM and periodontitis groups in oral samples were significant (*p* = 0.018, 0.016 and 0.021, respectively). There were no significant differences for the observed species index between healthy controls and the periodontitis group in either intestinal or oral samples (*p* > 0.05). The Shannon index for oral samples was significantly lower in healthy controls compared to the periodontitis or GDM group (*p* = 0.003 and 0.049, respectively). The Shannon index for intestinal samples was significantly lower in the periodontitis + GDM group in comparison with the periodontitis group (*p* = 0.004).

UPGMA analysis based on weighted UniFrac distances was performed to investigate the influence of GDM and periodontitis on the beta diversity of the oral and intestinal communities. From the results, all samples were separated into two groups with all oral samples in one cluster and all intestinal samples in the other cluster. Oral samples were sub-grouped into two clusters according to periodontal status, and groups with and without GDM were gathered together for both periodontitis and non-periodontitis conditions. With regard to the intestinal samples, two major clusters were found, with periodontitis + GDM in one group and healthy controls, periodontitis and GDM in the other group (Figure 3). The differences in microbial community structure among groups were also evaluated by AMOVA analysis (Table S3). Significant differences were found between healthy controls and the periodontitis group in oral samples (*p* < 0.001), while no significant differences between any other two groups in oral samples were found (*p*> 0.05). A significant difference in microbial structure was also found between the periodontitis + GDM group and healthy controls in intestinal samples (*p* = 0.043).

From the right part of Figure 3, obvious differences in composition of the top 10 phyla between oral and intestinal samples are demonstrated. The top 12 phyla of all samples were further compared among groups by MetaStat analysis (Figure S1). According to the results, the abundances of the top 4 phyla, *Proteobacteria, Firmicutes, Actinobacteria, and Bacteroidetes*, showed significant differences between any oral group and its corresponding intestinal group (*p* < 0.05). Lower *Firmicutes* levels and higher *Bacteroidetes* levels in the periodontitis + GDM group compared to healthy controls in intestinal samples were found, but the differences were not significant by MetaStat analysis (*p* > 0.05). In oral samples, the abundances of *Bacteroidetes, Spirochaetes, Tenericutes* and *Synergistetes* in the periodontitis group were significantly higher than those in healthy controls (*p* < 0.05).

To explore more details of the differences in the oral and intestinal microbiotas among the periodontitis, GDM, periodontitis + GDM and healthy control groups, LEfSe analysis was performed with an LDA value of 2.0. In oral samples, the highest LDA values were for s_*Porphyromonas_gingivalis* in the periodontitis group, f_*Ruminococcaceae* in the GDM group, s_*Bacteroides_eggerthii* in the periodontitis+GDM group and s_*Prevotella_sp* in the healthy control group (Figure 4(a)). In intestinal samples, the highest LDA values were for g_*Veillonella* in *the* periodontitis group, f_*Lachospiraceae* in the GDM group, *s-bacterium_enrichment_culture_clone_R4-81B* in the periodontitis + GDM group and s_Lactococcus_lactis in the healthy control group (Figure 4(b)). For the oral microbiota, the numbers of the differentially abundant taxa were 23 in the GDM group, 16 in the periodontitis group, 14 in healthy controls, and 3 in the periodontitis + GDM group respectively. Meanwhile, for the intestinal microbiota, the most enriched taxa number ranked as 38 in the GDM group, 23 in the periodontitis + GDM group, 8 in the periodontitis group, and 3 in healthy controls.

The cladograms display the differently expressed taxa among the four groups. The circle from inside to outside represents the classification levels from phyla to genera. The diameters of the circles represent relative abundances. At the family level, two oral taxa (*Desulfobulbaceae* and *Mycoplasmataceae*) and two intestinal taxa (*Atopobiaceae* and *Peptococcaceae*) in the periodontitis group, five oral taxa (*unidentified_Flavobacteriales, unidentified_Bacillales, Christensenellaceae, Ruminococcaceae* and *Enterobacteriaceae*) and three intestinal taxa (*Defluviitaleaceae, Lachnospiraceae* and *Paracaedibacteraceae*) in the GDM group, two intestinal taxa (*unidentified_Methanobacteriales* and *Nostocaceae*) in the periodontitis + GDM groups, four oral taxa (*Saprospiraceae, Microscillaceae, Rhodocyclaceae* and *Akkermansiaceae*) and one intestinal taxa (*Desulfobacteraceae*) in the healthy control group were significantly enriched (Figure 4 (c) and (d)).

**3. Analysis of the relationship between the oral and intestinal microbiotas and factors of the FBG levels and clinical parameters of periodontitis**

CCA analysis in phylum level was performed to investigate the relationship between microbial structures and clinical parameters of FBG levels, DMFT, meanPD and meanBI, as well as basic characteristics of age, preBMI and GWG. As showed in Figure 5, oral microbiota was obviously separated from the intestinal microbiota. A deviation trend was also found in oral microbiota between the periodontitis group and healthy controls. Among those maternal factors, FBG levels, meanBI and meanPD were detected to have significant effects on the microbial compositions by CCAenvfit analysis (*p* < 0.001, Table S4). FBG was oriented in the same direction as intestinal microbial distributions, while BI and PD were in the same orientations as oral microbial distributions. This might indicate a closer relationship between FBG and intestinal community distribution and between periodontal condition and oral community distribution among these three influencing factors.

**4. Microbial shifts between the oral and intestinal microbiotas under different periodontitis and GDM conditions**

**Figure 6.**
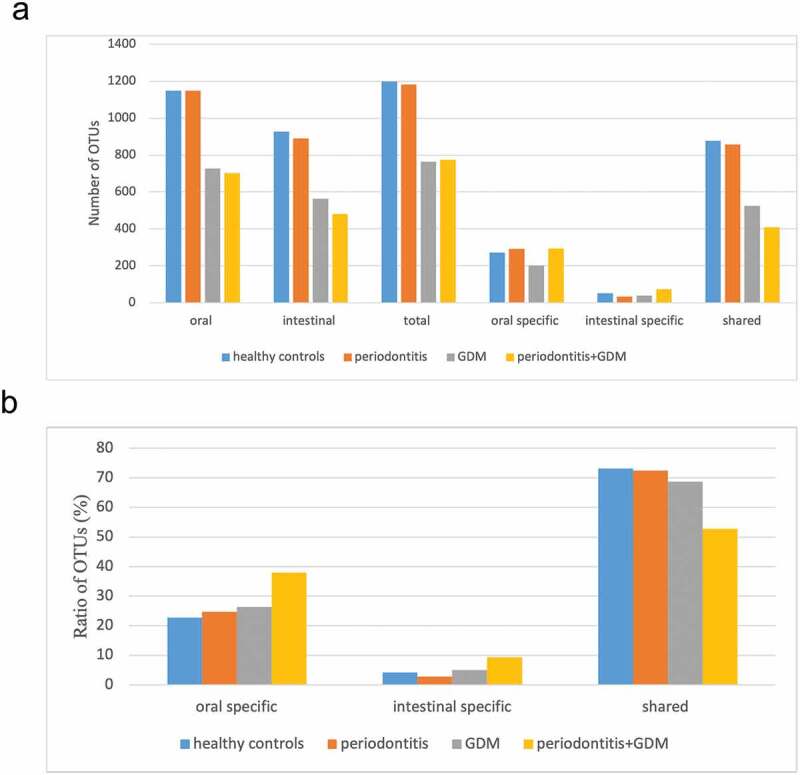
Comparisons of unique and shared OTU numbers (a) and ratios (b) at the genus level among different groups

To investigate whether GDM and periodontitis were associated with the shifts between the oral and intestinal microbiomes, the numbers and ratios of unique and shared OTUs in oral and intestinal samples were assessed (Figure S2 and Figure 6). From the results, compared to healthy controls, significant changes were found in cases with GDM. Abundances of all oral, intestinal and total OTUs were clearly reduced. Correspondingly, numbers of oral-specific, intestinal-specific and shared OTUs were lower. However, the proportions of oral-specific and intestinal-specific OTUs were both inversely elevated, and those of the shared OTUs were decreased. When GDM was coupled with periodontitis, a further decrease in oral and intestinal OTU numbers was found. However, both the numbers and percentages of oral- and intestinal-specific OTUs continued to increase even to the top of the four groups. Both numbers and percentages of the shared OTUs ranged at the lowest among the groups. In the case of periodontitis, the numbers of oral, intestinal and total OTUs were comparable to healthy controls, and both the numbers and percentages of oral-specific OTUs were increased, while intestinal-specific and shared OTUs were decreased (Figure 6).

## Discussion

The present study investigated the oral and intestinal microbiotas of middle-stage pregnant women with different periodontitis and GDM conditions. A significantly altered oral microbiota in the periodontitis group was observed, especially in subgingival plaques. When periodontitis was coupled with GDM, significant changes in intestinal microbial structure were found. The microbial shift in the oral and intestinal microbiotas was not obvious in the single periodontitis or GDM condition, and even ranked the least in the periodontitis + GDM group when measured by numbers and ratios of the shared OTUs. A separate influence of periodontitis and GDM on the oral and intestinal microbiotas may be indicated.

Saliva, supragingival plaque and subgingival plaque contain the most diverse microbiomes in the oral cavity and were mapped as different ecological niches [27,28]. From the results, significant differences in the overall microbial composition were shown among these three oral floras, especially between saliva and supragingival plaque. However, all oral samples overlapped with each other in a certain arranged sequence and clearly deviated from samples from the intestine. Preliminary examination of the relationship between diseases of GDM and periodontitis on community structure indicated a significant influence of periodontitis on subgingival microbiota, rather than on supragingival plaque and saliva. This was closely related to the nature and pathogenesis of periodontitis, which was biofilm initiated and characterized by attachment loss and deeper periodontal pocket formation and was consistent with previous studies [13,29].

A close relationship between pregnant periodontitis and oral microbiota was demonstrated by our results. Oral microbial evenness measured by the Shannon index was significantly higher in the periodontitis group than in the healthy control group. Oral microbial richness measured by observed species in the periodontitis group was also higher than that in healthy controls, although the differences were not significant. This was similar to findings for normal periodontitis, in which periodontal inflammation was associated with increased richness and diversity of the oral microbial community [13]. The more diverse community might represent a more stable ecosystem from the point of view of bacteria, and be the result of the destroyed host defenses associated with periodontitis [13]. Distinct oral community structure, especially in subgingival plaque from healthy controls, was also found in the periodontitis group in our study. The abundances of *Bacteroidetes, Spirochaetes, Tenericutes* and *Synergistetes* in the periodontitis group were significantly higher than those in the healthy control group. They were all Gram-negative, anaerobic, and previously identified as periodontitis-associated bacteria [13]. *Porphyromonas_gingivalis* was the dominant species among the differently expressed taxa in the periodontitis group (Figure 4(a)). All findings in oral microbiota of pregnant women with periodontitis were in accordance with those in normal subjects with periodontitis.

Apart from oral disease, oral microorganisms play important roles in various extraoral conditions [30]. Periodontopathic microorganisms such as *P. gingivalis* have been proven to be extensively associated with intestinal dysbiosis and proposed to constitute a possible pathway for periodontitis and systemic disease [10,31]. Lourenco et al. first compared intestinal microbial profiles among healthy individuals with gingivitis (N = 14) and periodontitis (N = 23) and periodontal healthy controls (N = 7) and reported a lower but not significant alpha diversity and an altered microbial composition in periodontitis patients [31]. In our study, no significant differences in Observed_species or Shannon index were found between pregnant women with and without periodontitis, although the periodontitis group possessed a lower median value of Observed_species index and a higher median value of Shannon index. The overall intestinal microbial compositions measured by weighted UniFrac distances and the levels of the phyla *Euryarchaeota, Verrucomicrobia* and *Proteobacteria* did not differ between the periodontitis group and the healthy control group. In addition to the pregnant state, race, dietary habit and limited sample size, discrepancy in defining periodontitis and controls might also contribute to the inconsistency between the two studies. In our study, non-periodontitis conditions, including gingivitis during pregnancy and gingival health, were merged into the non-periodontitis control group. Besides, plaque scores of all subjects were not evaluated in our study. Considering the fact that swallowing of high dose periodontopathic bacteria could induce a dysbiosis of the intestinal microbiota [11], it could not be excluded that the quantity of the swallowed periodontopathic microorganisms were too small to cause significant changes of intestinal microbiota. But interestingly, *veillonella*, which is an important opportunistic pathogen in the intestine and is associated with chronic inflammatory conditions and adverse pregnancy outcomes [32,33], was found to be significantly enriched in the periodontitis group compared to healthy controls (Figure 4(b)). Whether pregnant periodontitis is associated with dysbiosis of intestinal microbiome and whether the disturbance of intestinal microbiota constitutes a possible pathway directing periodontitis to pregnant health need to be further investigated.

Fourteen women in this study were diagnosed with GDM. Seven of them were also affected by periodontitis, and the other 7 were GDM only. The associations between the oral and intestinal microbiotas and GDM were also evaluated. From the results, when compared to the healthy controls, intestinal microbial richness and diversity measured by alpha diversity indexes were both lower in the GDM group, although the differences were not significant. This was consistent with the result of a recent published study by Chen T et al. They compared the gut microbial profiles of 110 GDM patients and 220 normal pregnant women in the second trimester and revealed a lower alpha diversity in GDM patients [34]. However, the abundance of *Lachnospiraceae*, which showed the richest levels in the GDM group in our study, was enriched in normoglycemic pregnant women. Similar to our results, in the studies of Cortez R V et al and Kuang Y S et al, *Lachnospiraceae* were found to be significantly increased in the GDM group and positively correlated with glucose tolerance [15,35]. Previously, an enrichment of intestinal *Lachnospiraceae* was also reported to be associated with metabolic disorders of type 2 diabetes, obesity and insulin resistance [36–38]. A causative effect of *Lachnospiraceae* strain on the induction of type 2 diabetes was revealed by Kameyam K et al. In their study, a strain of *Lachnospiraceae* (AJ110941) was isolated from the feces of hyperglycemic obese mice. Significant increases in fasting blood glucose levels as well as decreases in plasma insulin levels and HOMA-β values were found after the colonization of germ-free ob/ob mice by AJ110941 [39]. With regard to the oral microbiota, no significant changes in alpha diversity or beta diversity were found between the GDM group and the healthy control group. These were in agreement with findings of some previous studies, although inconsistent results exist [15–17]. *Ruminococcaceae*, one of the most typical gut microbiota taxa that has been reported to be elevated in cases of GDM [40], was also found to be significantly enriched in the oral microbiotas of GDM women in our study. A close relationship between *Ruminococcaceae* and glucose levels was indicated.

The relationship between periodontitis and GDM has been reported. The interplay between the oral and intestinal microbiotas was assumed to be another pathway linking periodontitis to general health [31]. Alterations of the oral and intestinal microbiomes by periodontitis or GDM alone were proposed to aggregate to some extent by the combination of the two diseases. In our study, significant changes in intestinal microbial richness (by observed species index) and structure (by weighted UniFrac distances) in the periodontitis + GDM group were found when compared with healthy controls. The abundances of *Lactococcus lactis* species and the *Desulfobacteraceae* family were decreased in the periodontitis and GDM groups, respectively, compared with healthy controls and were both further depressed in the periodontitis + GDM group (Figure S3 (a) and (b)). For the oral microbiota, community richness in the periodontitis + GDM group was significantly decreased compared with that in the healthy control group. When compared with healthy controls, *Bacteroides eggerthii* species were significantly enriched in the periodontitis group and GDM group and were enriched more in the periodontitis + GDM group (Figure S3 (c)). These consistent trends indicated the presence of a synergistic effect of periodontitis and GDM on the oral and intestinal microbiotas. However, the oral and intestinal microbial taxa that were mostly enriched in the periodontitis + GDM group did not exceed those of the diseased group and were even ranked the least in the oral microbiota. Independent roles of periodontitis plus GDM in the oral and intestinal microbiotas relative to both single conditions might be indicated by our study.

Microbial shifts in maternal microbiotas of different body sites have been reported to be associated with GDM. Microbiomes across body sites exhibited more similar structures in people with GDM than those without GDM, and the ratios of the OTUs shared by two or more sites were increased under GDM conditions [18]. Contrary to these findings, in our study, both the numbers and the ratios of OTUs shared by the oral and intestinal microbiomes were reduced in the GDM group and were further decreased when GDM was coupled with periodontitis. Whether there is a convergent tendency in microbiotas across body sites under diseased conditions such as GDM needs to be further explored.

Within the limitations of the study, a close relationship between pregnant periodontitis and oral microbiota was displayed in this study. Only a few similarities existed in the oral and intestinal microbial changes between those associated with periodontitis and those associated with GDM. Periodontitis combined with GDM might be a separate state that was different from each single disease condition in shaping the oral and intestinal microbiotas. Further studies with larger sample sizes need to be performed to verify the results.

## Supplementary Material

Supplemental Material
