# Supplementary material for: Differences in the oral and intestinal microbiotas in pregnant women varying in periodontitis and gestational diabetes mellitus conditions
Source: J Oral Microbiol. 2021 Feb 9;13(1):1883382. doi: 10.1080/20002297.2021.1883382 (PMC8676621; doi:10.1080/20002297.2021.1883382)
Supplement: Supplemental Material [file ZJOM_A_1883382_SM4171.zip › Supplementary files/Supplementary files (1).docx]

Table S1. AMOVA analysis between different groups based on weighted UniFrac distances.

|  | SS | df | MS | Fs | *p*-value |
| --- | --- | --- | --- | --- | --- |
| grouped by GDM |  |  |  |  |  |
| HA-HB | 3.470(19.291) | 1(149) | 3.470(0.129) | 26.802 | <0.001* |
| GA-GB | 0.971(4.375) | 1(36) | 0.971(0.122) | 7.991 | <0.001* |
| GC-HC | 0.104(4.274) | 1(67) | 0.104(0.064) | 1.630 | 0.141 |
| GA-GC | 1.440(1.817) | 1(26) | 1.440(0.070) | 20.597 | <0.001* |
| GB-HA | 1.820(8.063) | 1(77) | 1.820(0.105) | 17.381 | <0.001* |
| GB-HC | 3.957(6.832) | 1(77) | 3.957(0.089) | 44.600 | <0.001* |
| GC-HB | 3.241(15.611) | 1(108) | 3.241(0.145) | 22.422 | <0.001* |
| GB-HB | 0.043(18.169) | 1(118) | 0.043(0.154) | 0.282 | 0.992 |
| GB-GC | 2.415(4.382) | 1(36) | 2.415(0.122) | 19.842 | <0.001* |
| GA-HA | 0.050(5.498) | 1(67) | 0.050(0.082) | 0.611 | 0.740 |
| GA-HB | 1.239(15.604) | 1(108) | 1.239(0.144) | 8.575 | <0.001* |
| GA-HC | 2.136(4.267) | 1(67) | 2.136(0.064) | 33.540 | <0.001* |
| HA-HC | 5.700(7.955) | 1(108) | 5.700(0.074) | 77.387 | <0.001* |
| GC-HA | 2.563(5.505) | 1(67) | 2.563(0.082) | 31.189 | <0.001* |
| HB-HC | 7.941(18.061) | 1(149) | 7.941(0.121) | 65.509 | <0.001* |
| grouped by periodontitis | |  |  |  |  |
| A1-A2 | 0.098(5.450) | 1(67) | 0.098(0.081) | 1.203 | 0.240 |
| A2-B1 | 2.987(6.245) | 1(61) | 2.987(0.102) | 29.182 | <0.001* |
| B2-b2 | 2.577(7.165) | 1(64) | 2.577(0.112) | 23.013 | <0.001* |
| A1-B2 | 2.622(5.881) | 1(65) | 2.622(0.090) | 28.984 | <0.001* |
| C2-b2 | 3.297(6.144) | 1(66) | 3.297(0.093) | 35.416 | <0.001* |
| A2-C1 | 3.430(4.911) | 1(67) | 3.430(0.073) | 46.792 | <0.001* |
| A1-B1 | 2.762(5.866) | 1(60) | 2.72(0.098) | 28.247 | <0.001* |
| B1-C1 | 5.618(5.327) | 1(60) | 5.62(0.089) | 63.279 | <0.001* |
| B1-b1 | 0.897(6.849) | 1(52) | 0.897(0.132) | 6.811 | <0.001* |
| C1-b1 | 2.902(5.516) | 1(58) | 2.902(0.095) | 30.518 | <0.001* |
| B2-b1 | 0.764(6.864) | 1(57) | 0.764(0.120) | 6.348 | <0.001* |
| A1-b1 | 1.280(6.055) | 1(58) | 1.280(0.104) | 12.261 | <0.001* |
| B2-C2 | 5.443(5.668) | 1(66) | 5.443(0.086) | 63.383 | <0.001* |
| C2-b1 | 2.906(5.842) | 1(59) | 2.906(0.099) | 29.352 | <0.001* |
| A2-B2 | 2.756(6.259) | 1(66) | 2.756(0.095) | 29.063 | <0.001* |
| A1-C2 | 3.784(4.859) | 1(67) | 3.784(0.073) | 52.173 | <0.001* |
| A1-C1 | 3.942(4.533) | 1(66) | 3.942(0.069) | 57.403 | <0.001* |
| B1-B2 | 0.144(6.675) | 1(59) | 0.144(0.113) | 1.270 | 0.235 |
| A2-C2 | 3.226(5.238) | 1(68) | 3.226(0.077) | 41.881 | <0.001* |
| B1-b2 | 3.025(7.151) | 1(59) | 3.025(0.121) | 24.962 | <0.001* |
| A2-b1 | 1.228(6.433) | 1(59) | 1.228(0.109) | 11.263 | <0.001* |
| C1-b2 | 3.385(5.817) | 1(65) | 3.385(0.089) | 37.821 | <0.001* |
| A1-b2 | 2.068(6.356) | 1(65) | 2.068(0.098) | 21.150 | <0.001* |
| B2-C1 | 5.345(5.342) | 1(65) | 5.345(0.082) | 65.042 | <0.001* |
| A2-b2 | 1.772(6.735) | 1(66) | 1.771(0.102) | 17.363 | <0.001* |
| B1-C2 | 5.748(5.654) | 1(61) | 5.748(0.093) | 62.014 | <0.001* |
| b1-b2 | 0.794(7.340) | 1(57) | 0.794(0.129) | 6.166 | 0.001* |
| C1-C2 | 0.058(4.320) | 1(67) | 0.058(0.064) | 0.900 | 0.483 |

G and H represent samples with and without GDM, and numbers 2 and 1 represent samples with and without periodontitis, respectively. A, B, b and C represent salivary, supragingival, subgingival and intestinal samples, respectively. * indicates statistical significance (p < 0.05).

Table S2 Demographics and clinical status of the study population.

|  | periodontitis | GDM | periodontitis +GDM | Healthy controls | *P* value |
| --- | --- | --- | --- | --- | --- |
| N | 28 | 7 | 7 | 27 |  |
| Age (year) | 32.22(30.32-36.28) | 34.65(33.33-37.01) | 35.96(32.04-38.51) | 30.85(28.78-33.29) | 0.022 |
| preBMI (kg/m2) | 22.21(20.34-24.53) | 23.23(21.83-25.69) | 20.75(19.92-22.85) | 20.93(19.38-22.94) | 0.239 |
| GWG (kg) | 11(5-14) | 12(7.5-13) | 10.5(0-13.50) | 12(10-15) | 0.34 |
| meanBI | 2.75(2.51-3.43) | 1.45(1.35-1.92) | 2.85(2.40-3.35) | 1.40(1.10-1.85) | 0.000 |
| meanPD (mm) | 2.89(2.58-3.13) | 2.30(1.87-2.42) | 2.80(2.65-2.87) | 2.08(1.97-2.28) | 0.000 |
| DMFT | 2.50(0.25-4.75) | 2.00(0.00-5.00) | 3.00(0.00-7.00) | 2.00(1.00-8.00) | 0.726 |
| FBG level (mmol/L) | 4.65(4.35-4.80) | 4.70(4.60-4.80) | 5.00(4.70-5.20) | 4.50(4.30-4.60) | 0.014 |

preBMI: prepregnant BMI; GWG: gestational weight gain; MeanBI: the mean value of gingival bleeding index; meanPD: the mean value of periodontal probing depth; DMFT: decayed, missing, and filled teeth; FBG: fasting blood glucose.

Values represent the median with the interquartile range. Significant differences were determined by the Mann-Whitney U-test.

Table S3. AMOVA analysis among different groups based on weighted UniFrac distances when oral samples were grouped together.

| Groups | SS | df | MS | Fs | p-value |
| --- | --- | --- | --- | --- | --- |
| HC1-HC2 | 0.033(3.330) | 1(53) | 0.033(0.063) | 0.520 | 0.831 |
| GC1-GC2 | 0.089(0.823) | 1(12) | 0.089(0.069) | 1.297 | 0.25 |
| GC1-GO2 | 1.246(3.493) | 1(26) | 1.246(0.134) | 9.274 | <0.001* |
| GO2-HO1 | 0.196(13.836) | 1(90) | 0.196(0.154) | 1.277 | 0.232 |
| GO1-GO2 | 0.106(5.242) | 1(36) | 0.106(0.146) | 0.727 | 0.64 |
| GC2-HC2 | 0.152(2.172) | 1(33) | 0.152(0.066) | 2.305 | 0.051 |
| GO2-HO2 | 0.082(14.725) | 1(99) | 0.082(0.149) | 0.554 | 0.804 |
| GO1-HO1 | 0.102(12.657) | 1(86) | 0.102(0.147) | 0.690 | 0.701 |
| GC2-GO1 | 1.113(2.573) | 1(22) | 1.113(0.117) | 9.519 | <0.001* |
| GC1-HO2 | 1.340(11.796) | 1(85) | 1.340(0.139) | 9.654 | <0.001* |
| HC1-HO2 | 3.689(13.214) | 1(105) | 3.689(0.126) | 29.313 | <0.001* |
| GO1-HO2 | 0.149(13.545) | 1(95) | 0.149(0.143) | 1.047 | 0.353 |
| GC2-HO2 | 1.399(12.056) | 1(85) | 1.399(0.142) | 9.863 | <0.001* |
| GC1-GO1 | 1.069(2.313) | 1(22) | 1.069(0.105) | 10.168 | <0.001* |
| GO1-HC1 | 1.986(3.731) | 1(42) | 1.986(0.089) | 22.356 | <0.001* |
| HC2-HO1 | 4.510(12.256) | 1(97) | 4.510(0.126) | 35.698 | <0.001* |
| GO1-HC2 | 1.947(3.662) | 1(43) | 1.947(0.085) | 22.860 | <0.001* |
| GC2-GO2 | 1.287(3.752) | 1(26) | 1.287(0.144) | 8.914 | <0.001* |
| GC1-HC1 | 0.016(1.981) | 1(32) | 0.016(0.062) | 0.253 | 0.997 |
| HO1-HO2 | 0.622(22.139) | 1(149) | 0.622(0.149) | 4.184 | <0.001* |
| GC1-HO1 | 1.667(10.907) | 1(76) | 1.667(0.142) | 11.616 | <0.001* |
| HC1-HO1 | 4.466(12.325) | 1(96) | 4.466(0.128) | 34.787 | <0.001* |
| GO2-HC1 | 2.473(4.910) | 1(46) | 2.473(0.107) | 23.162 | <0.001* |
| GC2-HO1 | 1.773(11.167) | 1(76) | 1.773(0.147) | 12.067 | <0.001* |
| GC1-HC2 | 0.031(1.912) | 1(33) | 0.031(0.058) | 0.536 | 0.759 |
| GO2-HC2 | 2.445(4.842) | 1(47) | 2.445(0.103) | 23.732 | <0.001* |
| GC2-HC1 | 0.164(2.241) | 1(32) | 0.164(0.070) | 2.340 | 0.043 |
| HC2-HO2 | 3.655(13.145) | 1(106) | 3.655(0.124) | 29.470 | <0.001* |

G and H represent samples with and without GDM, and numbers 2 and 1 represent samples with and without periodontitis, respectively. O and C represent oral and intestinal samples, respectively. * indicates statistical significance (p < 0.05).

Table S4 Envfit fits the environmental factors onto canonical correlation analysis (CCA) ordination plots.

|  | CCA1 | CCA2 | r2 | Pr(>r) |
| --- | --- | --- | --- | --- |
| Age | 0.535 | 0.845 | 0.007 | 0.396 |
| preBMI | -0.647 | 0.762 | 0.004 | 0.587 |
| GWG | -0.436 | -0.900 | 0.020 | 0.079 |
| FBG | -0.625 | 0.781 | 0.056 | 0.001 |
| MeanPD | 0.990 | 0.142 | 0.067 | 0.000 |
| MeanBI | 0.933 | 0.360 | 0.064 | 0.000 |
| DMFT | 0.968 | -0.251 | 0.002 | 0.750 |

Figure legends

**Figure S1** Comparisons of the TOP12 phyla among different groups by MetaStat analysis. G indicates GDM+, H indicates GDM-, 2 and 1 indicate with and without periodontitis, respectively. C and O represent the gut and oral microbiomes, respectively. *indicates *p*<0.05, **indicates *p*<0.01

**Figure S2** Venn diagrams of unique and shared OTU numbers at genus levels in each oral-intestinal paired group. G indicates GDM+, H indicates GDM-, 2 and 1 indicate with and without periodontitis, respectively. C and O represent the gut and oral microbiomes, respectively.

**Figure S3** Comparisons of the relative abundances of *Lactococcus lactis* species, *Desulfobacteraceae* family, *and Bacteroides eggerthii* species among groups. The bar graph indicates the individual sample, the vertical axis indicates the relative abundance, the horizontal lines indicate the mean value of the abundance. G indicates GDM+, H indicates GDM-, 2 and 1 indicate with and without periodontitis, respectively. C and O represent the gut and oral microbiomes, respectively.
