# Supplementary figures and images for: Differences in the oral and intestinal microbiotas in pregnant women varying in periodontitis and gestational diabetes mellitus conditions
Source: J Oral Microbiol. 2021 Feb 9;13(1):1883382. doi: 10.1080/20002297.2021.1883382 (PMC8676621; doi:10.1080/20002297.2021.1883382)

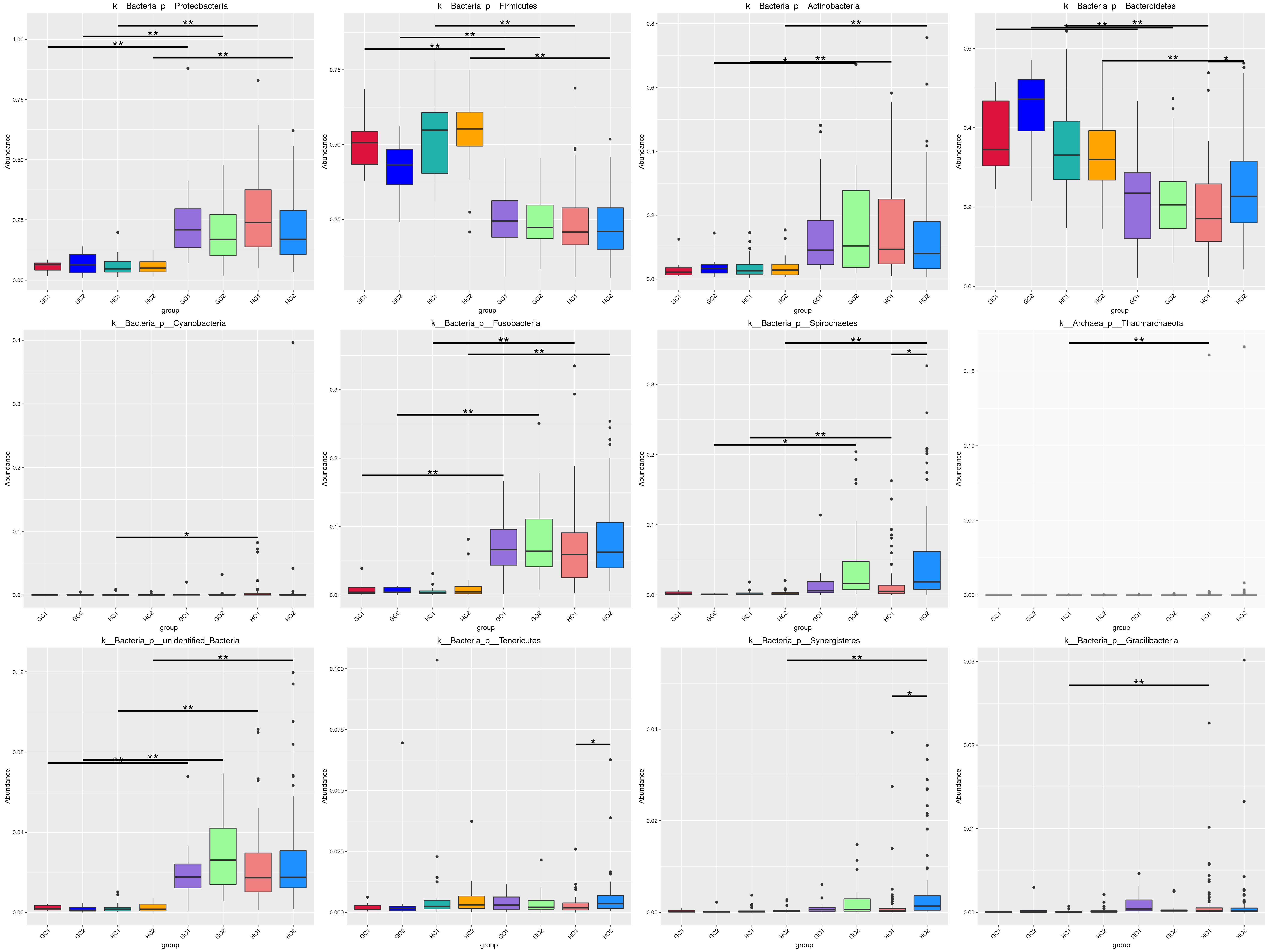

Supplement: Supplemental Material [file ZJOM_A_1883382_SM4171.zip › Supplementary files/Figure S1.png]

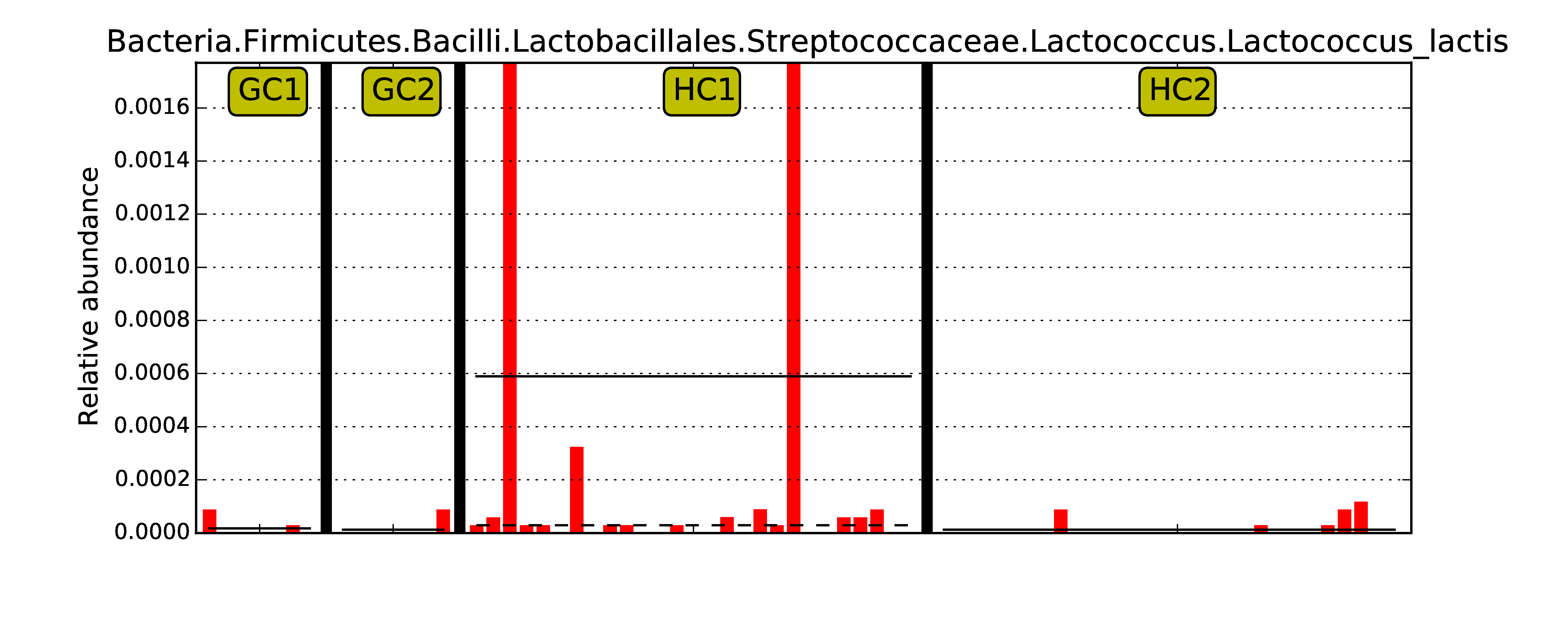

Supplement: Supplemental Material [file ZJOM_A_1883382_SM4171.zip › Supplementary files/Figure S3(a).png]

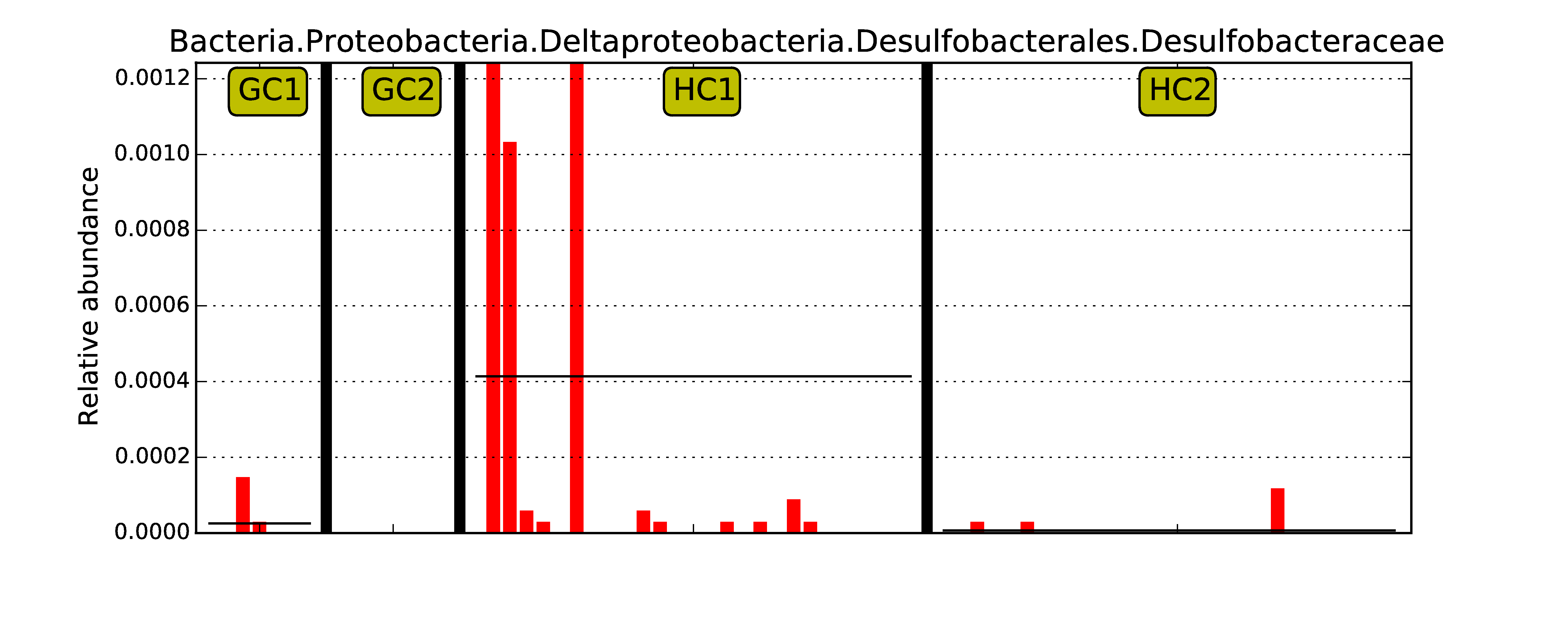

Supplement: Supplemental Material [file ZJOM_A_1883382_SM4171.zip › Supplementary files/Figure S3(b).png]

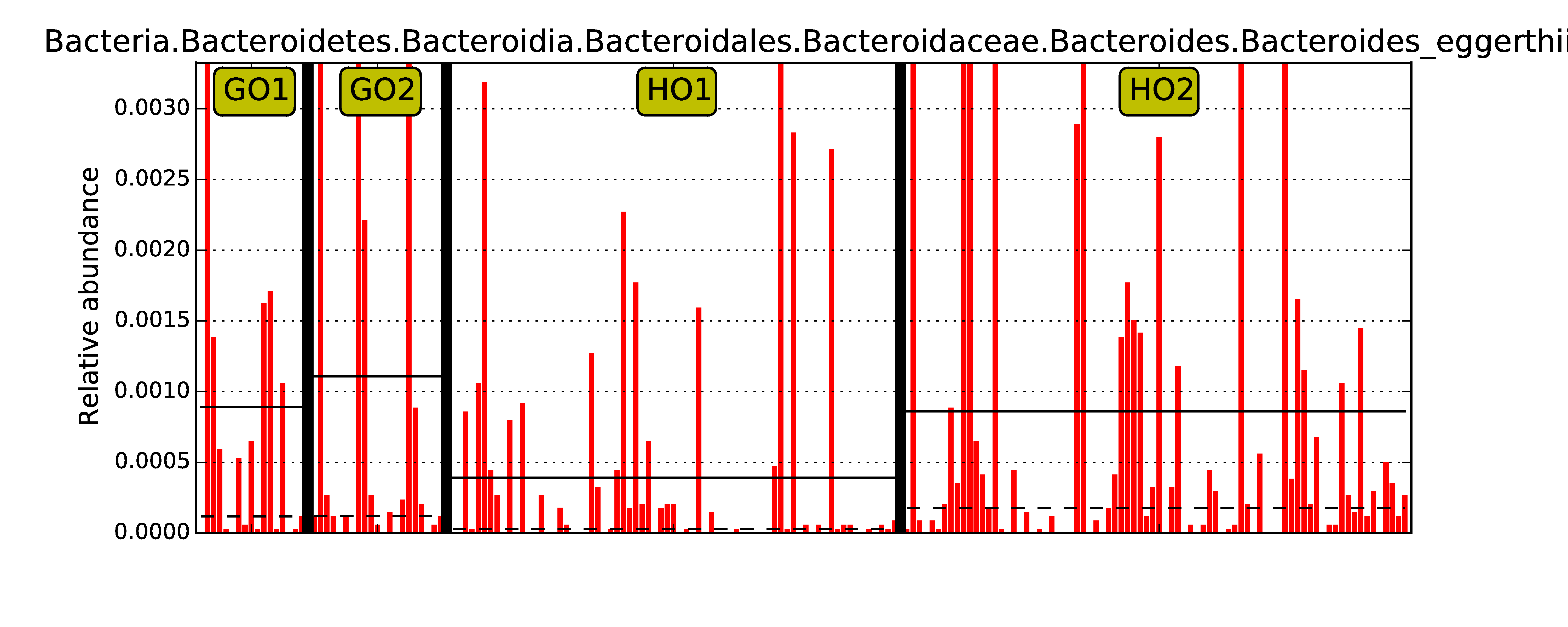

Supplement: Supplemental Material [file ZJOM_A_1883382_SM4171.zip › Supplementary files/Figure S3(c).png]
